# *N*-[(*E*)-Quinoxalin-2-ylmethyl­idene]-1*H*-indazol-5-amine

**DOI:** 10.1107/S1600536809027822

**Published:** 2009-07-25

**Authors:** P. Leeju, V. Arun, Manju Sebastian, G. Varsha, Digna Varghese, K. K. M. Yusuff

**Affiliations:** a Department of Applied Chemistry, Cochin University of Science and Technology, Cochin 682 022, Kerala, India

## Abstract

In the title mol­ecule, C_16_H_11_N_5_, the mean planes of the quinoxaline and indazole fragments form a dihedral angle of 10.62 (5)°. In the crystal, weak inter­molecular N—H⋯N hydrogen bonds link the mol­ecules into zigzag chains extending in the [001] direction. The crystal packing also exhibits π–π inter­actions [centroid–centroid distances of 3.7080 (2) and 3.8220 (5) Å], which form stacks of the mol­ecules parallel to the *a* axis.

## Related literature

For related structures, see: Varghese *et al.* (2009 ▶); Varsha *et al.* (2009 ▶).
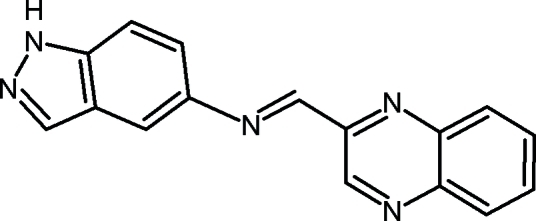


## Experimental

### 

#### Crystal data

C_16_H_11_N_5_
*M* *_r_* = 273.30
Monoclinic, 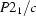

*a* = 7.7015 (6) Å
*b* = 8.0330 (6) Å
*c* = 20.6034 (16) Å
β = 96.882 (2)°
*V* = 1265.47 (17) Å^3^
*Z* = 4
Mo *K*α radiation
μ = 0.09 mm^−1^
*T* = 298 K
0.45 × 0.27 × 0.08 mm

#### Data collection

Bruker Kappa APEX CCD diffractometer
Absorption correction: multi-scan (*SADABS*; Sheldrick, 2001 ▶) *T*_min_ = 0.960, *T*_max_ = 0.993
16012 measured reflections
3597 independent reflections
2502 reflections with *I* > 2σ(*I*)
*R*_int_ = 0.024

#### Refinement

*R*[*F* ^2^ > 2σ(*F* ^2^)] = 0.046
*wR*(*F* ^2^) = 0.143
*S* = 1.03
3597 reflections
190 parameters
H-atom parameters constrained
Δρ_max_ = 0.23 e Å^−3^
Δρ_min_ = −0.26 e Å^−3^

### 

Data collection: *SMART* (Bruker, 2000 ▶); cell refinement: *SAINT* (Bruker, 2000 ▶); data reduction: *SAINT*; program(s) used to solve structure: *SHELXS97* (Sheldrick, 2008 ▶); program(s) used to refine structure: *SHELXL97* (Sheldrick, 2008 ▶); molecular graphics: *SHELXTL* (Sheldrick, 2008 ▶); software used to prepare material for publication: *publCIF* (Westrip, 2009 ▶).

## Supplementary Material

Crystal structure: contains datablocks I, global, schiflm. DOI: 10.1107/S1600536809027822/cv2578sup1.cif

Structure factors: contains datablocks I. DOI: 10.1107/S1600536809027822/cv2578Isup2.hkl

Additional supplementary materials:  crystallographic information; 3D view; checkCIF report

## Figures and Tables

**Table 1:** Hydrogen-bond geometry (Å, °)

*D*—H⋯*A*	*D*—H	H⋯*A*	*D*⋯*A*	*D*—H⋯*A*
N4—H4⋯N1^iii^	0.86	2.31	3.1050 (15)	153

Symmetry code: (iii) 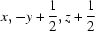.

**Fig. 1.**
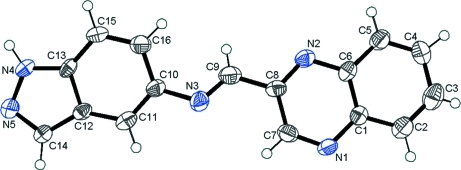
The molecular structure of (I), showing the atom-numbering scheme. Displacement ellipsoids are drawn at the 50% probability level.

## supplementary crystallographic information

### Comment

In view of synthesizing new quinoxaline based Schiff bases, we have undertaken the synthesis of the title compound, (1), and report here its crystal structure. In (1), the quinoxaline ring and indazole ring are each approximately planar, with the maximum deviations of 0.0254 (4) and 0.0213 (4) Å from the least square planes, respectively. A perspective drawing is depicted in figure 1 with the atomic numbering scheme. The compound is non-planar due to the twisting of rings with respect to azomethine group. Bond lengths and angles are in normal ranges and comparable to those in related structures (Varghese *et al.*, 2009; Varsha *et al.*, 2009). In the crystal structure, molecules are held together by π–π stacking interactions and N—H···N intermolecular hydrogen bonding.

### Experimental

A hot solution of 5-aminoindazole (1 mmol) in ethanol (20 ml) was added slowly to a hot solution of quinoxaline-2-carboxaldehyde (1 mmol) in the same solvent (40 ml).The resulting mixture on cooling yielded the crude product of (1). Pale green crystals suitable for single-crystal XRD are obtained by slow evaporation of ethanolic solution of (1).

### Refinement

H atoms were positioned geometrically (N—H = 0.86 Å, C—H = 0.93 Å) and refined in riding mode, with *U*_iso_ (H) = 1.2Ueq(C, N).

### Crystal data

**Table:**

C_16_H_11_N_5_	*F*(000) = 568
*M**_r_* = 273.30	*D*_x_ = 1.434 Mg m^−^^3^
Monoclinic, *P*2_1_/*c*	Mo *K*α radiation, λ = 0.71073 Å
Hall symbol: -P 2ybc	Cell parameters from 2502 reflections
*a* = 7.7015 (6) Å	θ = 2.6–29.8°
*b* = 8.0330 (6) Å	µ = 0.09 mm^−^^1^
*c* = 20.6034 (16) Å	*T* = 298 K
β = 96.882 (2)°	Plate, green
*V* = 1265.47 (17) Å^3^	0.45 × 0.27 × 0.08 mm
*Z* = 4	

### Data collection

**Table:**

Bruker Kappa APEX CCD diffractometer	3597 independent reflections
Radiation source: fine-focus sealed tube	2502 reflections with *I* > 2σ(*I*)
graphite	*R*_int_ = 0.024
ω and φ scans	θ_max_ = 29.8°, θ_min_ = 2.7°
Absorption correction: multi-scan (*SADABS*; Sheldrick, 2001)	*h* = −10→10
*T*_min_ = 0.960, *T*_max_ = 0.993	*k* = −11→10
16012 measured reflections	*l* = −28→26

### Refinement

**Table:**

Refinement on *F*^2^	Primary atom site location: structure-invariant direct methods
Least-squares matrix: full	Secondary atom site location: difference Fourier map
*R*[*F*^2^ > 2σ(*F*^2^)] = 0.046	Hydrogen site location: inferred from neighbouring sites
*wR*(*F*^2^) = 0.143	H-atom parameters constrained
*S* = 1.03	*w* = 1/[σ^2^(*F*_o_^2^) + (0.0715*P*)^2^ + 0.2171*P*] where *P* = (*F*_o_^2^ + 2*F*_c_^2^)/3
3597 reflections	(Δ/σ)_max_ = 0.003
190 parameters	Δρ_max_ = 0.23 e Å^−^^3^
0 restraints	Δρ_min_ = −0.26 e Å^−^^3^

### Special details

**Table:**

Geometry. All e.s.d.'s (except the e.s.d. in the dihedral angle between two l.s. planes) are estimated using the full covariance matrix. The cell e.s.d.'s are taken into account individually in the estimation of e.s.d.'s in distances, angles and torsion angles; correlations between e.s.d.'s in cell parameters are only used when they are defined by crystal symmetry. An approximate (isotropic) treatment of cell e.s.d.'s is used for estimating e.s.d.'s involving l.s. planes.
Refinement. Refinement of *F*^2^ against ALL reflections. The weighted *R*-factor *wR* and goodness of fit *S* are based on *F*^2^, conventional *R*-factors *R* are based on *F*, with *F* set to zero for negative *F*^2^. The threshold expression of *F*^2^ > σ(*F*^2^) is used only for calculating *R*-factors(gt) *etc*. and is not relevant to the choice of reflections for refinement. *R*-factors based on *F*^2^ are statistically about twice as large as those based on *F*, and *R*- factors based on ALL data will be even larger.

### Fractional atomic coordinates and isotropic or equivalent isotropic displacement parameters (Å^2^)

**Table:**

	*x*	*y*	*z*	*U*_iso_*/*U*_eq_	
N1	0.25032 (15)	0.66502 (15)	0.33833 (5)	0.0436 (3)	
N2	0.14194 (13)	0.77204 (14)	0.45776 (5)	0.0392 (3)	
N3	0.30313 (15)	0.37642 (14)	0.50779 (5)	0.0413 (3)	
N4	0.38605 (15)	−0.08636 (16)	0.70573 (5)	0.0454 (3)	
H4	0.3774	−0.0851	0.7470	0.054*	
N5	0.42455 (17)	−0.22452 (16)	0.67229 (6)	0.0515 (3)	
C1	0.18211 (15)	0.82051 (17)	0.34459 (6)	0.0368 (3)	
C2	0.16182 (18)	0.9289 (2)	0.29041 (6)	0.0469 (3)	
H2	0.1980	0.8960	0.2509	0.056*	
C3	0.08916 (19)	1.0817 (2)	0.29612 (7)	0.0507 (4)	
H3	0.0724	1.1511	0.2598	0.061*	
C4	0.03910 (19)	1.1364 (2)	0.35576 (7)	0.0490 (4)	
H4A	−0.0073	1.2426	0.3589	0.059*	
C5	0.05790 (18)	1.03530 (18)	0.40912 (7)	0.0440 (3)	
H5	0.0247	1.0724	0.4486	0.053*	
C6	0.12780 (16)	0.87452 (17)	0.40443 (6)	0.0361 (3)	
C7	0.26273 (18)	0.57069 (18)	0.39027 (6)	0.0436 (3)	
H7	0.3088	0.4642	0.3877	0.052*	
C8	0.20879 (16)	0.62367 (16)	0.45062 (6)	0.0374 (3)	
C9	0.22517 (17)	0.51440 (18)	0.50816 (6)	0.0411 (3)	
H9	0.1773	0.5475	0.5455	0.049*	
C10	0.32246 (16)	0.27137 (17)	0.56339 (6)	0.0369 (3)	
C11	0.36804 (16)	0.10932 (16)	0.55175 (6)	0.0375 (3)	
H11	0.3848	0.0756	0.5098	0.045*	
C12	0.38887 (16)	−0.00418 (17)	0.60359 (6)	0.0360 (3)	
C13	0.36273 (15)	0.04951 (17)	0.66654 (6)	0.0365 (3)	
C14	0.42856 (19)	−0.17552 (18)	0.61149 (6)	0.0460 (3)	
H14	0.4543	−0.2451	0.5778	0.055*	
C15	0.32249 (17)	0.21454 (18)	0.67939 (6)	0.0421 (3)	
H15	0.3096	0.2496	0.7216	0.051*	
C16	0.30258 (18)	0.32333 (18)	0.62789 (6)	0.0427 (3)	
H16	0.2754	0.4339	0.6354	0.051*	

### Atomic displacement parameters (Å^2^)

**Table:**

	*U*^11^	*U*^22^	*U*^33^	*U*^12^	*U*^13^	*U*^23^
N1	0.0514 (6)	0.0477 (7)	0.0324 (5)	0.0016 (5)	0.0079 (4)	−0.0037 (5)
N2	0.0438 (6)	0.0433 (6)	0.0313 (5)	0.0018 (5)	0.0081 (4)	0.0014 (4)
N3	0.0508 (6)	0.0404 (6)	0.0333 (5)	−0.0004 (5)	0.0073 (4)	0.0023 (4)
N4	0.0577 (7)	0.0510 (7)	0.0279 (5)	−0.0001 (5)	0.0070 (5)	0.0050 (5)
N5	0.0698 (8)	0.0469 (7)	0.0377 (6)	0.0017 (6)	0.0070 (5)	0.0050 (5)
C1	0.0357 (6)	0.0445 (7)	0.0304 (6)	−0.0040 (5)	0.0046 (4)	−0.0005 (5)
C2	0.0492 (7)	0.0593 (9)	0.0333 (6)	−0.0023 (7)	0.0096 (5)	0.0060 (6)
C3	0.0494 (8)	0.0581 (9)	0.0453 (8)	−0.0007 (7)	0.0087 (6)	0.0184 (7)
C4	0.0473 (7)	0.0466 (8)	0.0536 (8)	0.0046 (6)	0.0085 (6)	0.0093 (7)
C5	0.0465 (7)	0.0465 (8)	0.0398 (7)	0.0042 (6)	0.0087 (5)	0.0011 (6)
C6	0.0355 (6)	0.0423 (7)	0.0307 (6)	−0.0030 (5)	0.0046 (4)	0.0000 (5)
C7	0.0530 (8)	0.0435 (8)	0.0347 (6)	0.0041 (6)	0.0065 (5)	−0.0026 (5)
C8	0.0401 (6)	0.0402 (7)	0.0322 (6)	−0.0015 (5)	0.0061 (5)	0.0006 (5)
C9	0.0452 (7)	0.0452 (8)	0.0338 (6)	0.0000 (6)	0.0087 (5)	0.0022 (5)
C10	0.0417 (6)	0.0405 (7)	0.0291 (6)	−0.0015 (5)	0.0071 (5)	0.0001 (5)
C11	0.0440 (6)	0.0435 (7)	0.0264 (5)	0.0002 (5)	0.0098 (5)	−0.0015 (5)
C12	0.0395 (6)	0.0406 (7)	0.0286 (6)	−0.0015 (5)	0.0067 (4)	−0.0007 (5)
C13	0.0367 (6)	0.0468 (8)	0.0264 (5)	−0.0026 (5)	0.0054 (4)	0.0012 (5)
C14	0.0604 (8)	0.0425 (8)	0.0356 (7)	0.0029 (6)	0.0075 (6)	0.0008 (6)
C15	0.0489 (7)	0.0516 (8)	0.0265 (6)	0.0014 (6)	0.0074 (5)	−0.0063 (5)
C16	0.0531 (7)	0.0420 (7)	0.0334 (6)	0.0038 (6)	0.0062 (5)	−0.0063 (5)

### Geometric parameters (Å, °)

**Table:**

N1—C7	1.3053 (17)	C5—C6	1.4070 (19)
N1—C1	1.3670 (17)	C5—H5	0.9300
N2—C8	1.3136 (17)	C7—C8	1.4225 (17)
N2—C6	1.3668 (16)	C7—H7	0.9300
N3—C9	1.2610 (18)	C8—C9	1.4684 (17)
N3—C10	1.4162 (16)	C9—H9	0.9300
N4—C13	1.3568 (17)	C10—C11	1.3768 (18)
N4—N5	1.3576 (17)	C10—C16	1.4185 (17)
N4—H4	0.8600	C11—C12	1.3989 (17)
N5—C14	1.3169 (17)	C11—H11	0.9300
C1—C2	1.4094 (18)	C12—C13	1.4039 (16)
C1—C6	1.4167 (17)	C12—C14	1.4150 (19)
C2—C3	1.360 (2)	C13—C15	1.3941 (19)
C2—H2	0.9300	C14—H14	0.9300
C3—C4	1.402 (2)	C15—C16	1.3691 (18)
C3—H3	0.9300	C15—H15	0.9300
C4—C5	1.3605 (19)	C16—H16	0.9300
C4—H4A	0.9300		
			
Cg1···Cg3^i^	3.7080 (2)	Cg2···Cg3^ii^	3.8220 (5)
			
C7—N1—C1	116.38 (11)	N2—C8—C7	121.92 (12)
C8—N2—C6	116.81 (10)	N2—C8—C9	116.67 (11)
C9—N3—C10	121.52 (11)	C7—C8—C9	121.40 (12)
C13—N4—N5	112.15 (10)	N3—C9—C8	121.03 (12)
C13—N4—H4	123.9	N3—C9—H9	119.5
N5—N4—H4	123.9	C8—C9—H9	119.5
C14—N5—N4	105.65 (12)	C11—C10—N3	115.26 (11)
N1—C1—C2	119.81 (11)	C11—C10—C16	119.99 (12)
N1—C1—C6	121.24 (11)	N3—C10—C16	124.73 (12)
C2—C1—C6	118.94 (12)	C10—C11—C12	119.44 (11)
C3—C2—C1	119.78 (13)	C10—C11—H11	120.3
C3—C2—H2	120.1	C12—C11—H11	120.3
C1—C2—H2	120.1	C11—C12—C13	119.28 (12)
C2—C3—C4	121.20 (13)	C11—C12—C14	136.49 (12)
C2—C3—H3	119.4	C13—C12—C14	104.21 (11)
C4—C3—H3	119.4	N4—C13—C15	131.95 (11)
C5—C4—C3	120.49 (14)	N4—C13—C12	106.21 (12)
C5—C4—H4A	119.8	C15—C13—C12	121.83 (12)
C3—C4—H4A	119.8	N5—C14—C12	111.77 (12)
C4—C5—C6	119.80 (13)	N5—C14—H14	124.1
C4—C5—H5	120.1	C12—C14—H14	124.1
C6—C5—H5	120.1	C16—C15—C13	117.81 (11)
N2—C6—C5	119.47 (11)	C16—C15—H15	121.1
N2—C6—C1	120.78 (12)	C13—C15—H15	121.1
C5—C6—C1	119.75 (12)	C15—C16—C10	121.58 (13)
N1—C7—C8	122.85 (13)	C15—C16—H16	119.2
N1—C7—H7	118.6	C10—C16—H16	119.2
C8—C7—H7	118.6		

Symmetry codes: (i) −*x*, −*y*+2, −*z*; (ii) −*x*+2, −*y*+2, −*z*+2.

### Hydrogen-bond geometry (Å, °)

**Table:**

*D*—H···*A*	*D*—H	H···*A*	*D*···*A*	*D*—H···*A*
N4—H4···N1^iii^	0.86	2.31	3.1050 (15)	153

Symmetry codes: (iii) *x*, −*y*+1/2, *z*+1/2.
